# The forgotten facet of firearms safety: Unintentional gun injuries and deaths in the United States

**DOI:** 10.3389/fpubh.2023.1137725

**Published:** 2023-03-23

**Authors:** David C. Schwebel

**Affiliations:** Department of Psychology, University of Alabama at Birmingham, Birmingham, AL, United States

**Keywords:** firearms, gun injuries, unintentional injuries, safety, United States, health behavior change

## Introduction

Firearm-related injuries and deaths are endemic in the United States today. Reading the daily news, many American citizens reflect upon the horrors of mass shootings and sense the daily homicides and suicides that kill 5 Americans every hour of every day, on average (1).

Less publicized but equally tragic are unintentional firearm-related deaths and injuries. Validating the accuracy of data remains challenging (2), but CDC estimates suggest that in 2020, about 535 Americans were killed by an “accidental” firearm-related death. Approximately 29,000 were injured seriously enough to visit the emergency department. Over 25% of those deaths and 15% of injuries were children (1).

Strategies to prevent unintentional firearms-related injuries and deaths overlap with but are unique from those to prevent suicide and homicide. Policy-making is discussed most prominently. Laws that keep guns out of the hands of youth, criminals, and those with serious mental illness, for example, will likely prevent unintentional firearms injuries as well as intentional ones. However, they are unlikely to create major change. Guns are and will likely always remain in American homes, suggesting they are and will likely always remain a threat for unintentional misuse.

## Solutions

Three concurrent strategies might slow the scourge of unintentional firearms injuries: change adult behavior, change youth behavior, and engineer safer firearm and firearm storage options. Pervading all three is the need for respect and cooperation with firearms owners. Almost universally, gun owners support firearms safety. They readily acknowledge the potential of their firearms to cause injury and they support efforts to teach safety. The Public Health Code of Ethics mandates cultural sensitivity and inclusivity by public health professionals, principles that apply not just to underserved or minority groups but also to sociopolitical groups like firearms owners who voice firm opinions about policies and legislation related to firearms ownership and use (3).

### Change adult behavior

Human health behaviors are resistant to change. Most people who smoke cigarettes recognize smoking is bad for their health, but they don't necessarily quit. Individuals with obesity often recognize they should lose weight but don't consistently take steps to do so. Similarly, most firearms owners pride themselves in maintaining safety but may occasionally slip into risky behaviors. They may unknowingly leave a gun loaded when distracted by an urgent phone call. They may face unexpectedly cold weather while hunting, unthinkingly don a jacket over their hunter's orange vest, and place themselves at camouflaged risk. They may return home after a long and stressful day of work and drop a loaded occupation-related firearm in the front hallway to be discovered by a toddler. In each case, “slips” can lead to tragedies.

Prevention requires the full arsenal of public health theory and practice to create culturally-accepted behavior change. Theory-driven, empirically-supported interventions can change behavior, alter habits, and create constant and unwavering attention to safety. The logical vehicle to deliver such interventions is through wide dissemination of high-quality firearms safety training that is empirically supported to increase safe firearms handling and storage among attendees.

Non-occupational firearms safety courses involving experiential training on ranges are widely available, but can be expensive. In a 2019 report on the topic, training courses lasted a mean of 6 h and cost an average of $130 (4). Accessibility and affordability would improve delivery of safety lessons. As an example, the State of Alabama Department of Conservation and Natural Resources recently introduced firearms safety courses for the public that cost just $12, offering an affordable way for citizens to learn key safety lessons in the context of live shooting. Other states and agencies might consider such initiatives.

Also critical is the content of courses. Available evidence suggests almost all courses cover key topics like keeping your finger off the trigger until ready to shoot, pointing the muzzle in safe directions, and checking your target and what is beyond (4), but they omit topics that may be of less interest to their audience or outside the expertise of the instructors, such as suicide and domestic violence prevention (4). There also is little published evidence indicating existing courses are based in social science theory designed to change safety behaviors. Consideration of how to develop, evaluate, and provide firearms safety instruction from a behavioral science perspective, including delivery of theory-driven lessons that concord with epidemiological data on the greatest risks and successfully convey key safety-promoting messages to firearm-owning adults in a manner compatible with the political and sociocultural views of the likely audience, could improve safety.

### Change youth behavior

Some American children learn to hunt and shoot before they learn to read; blogger mom Joyce Wild wrote, “My children have been hunting since they were in diapers” (5). The National Rifle Association suggests children may be ready to learn to shoot between the ages of 6 and 8 (6). Published evidence suggests about one-third of American youth live in homes with firearms (7) and children routinely learn to hunt and shoot during the early elementary school years (8).

Thus, efforts to argue children should never touch or live near guns are likely to be futile. Instead, we must use public health theory and data to develop strategies to teach children firearms safety. Young children, roughly those under age 10, might be taught to touch and use firearms only under adult supervision. By age 10, children are developing the cognitive capacity to use firearms safely. At that point, theory-driven educational programs could guide them in safe firearm handling and storage (9). As in all public health domains, including firearms safety training for adults, the need for theory-driven and empirically-supported interventions targeting the greatest risks is foremost. Current evidence is sparse, as most existing child firearms safety training programs are untested in rigorous scientific trials. Evaluations of programs that have been conducted yield mixed results (10, 11). Efforts to develop and evaluate theory-based, child-oriented firearms safety programs are therefore urgently needed. Replicating adult training courses, they must be delivered in a way that is sensitive to the political and sociocultural views of the likely audience, and they must be based in theory-driven mechanisms to achieve behavior change. Use of ineffective or unproven educational programs is unproductive and potentially detrimental, as it inaccurately implies to youth and their parents that the children have learned safety.

### Engineer safer firearm design and storage options

Innovative engineering transformed fields like traffic safety (12) and occupational safety (13). Parallel efforts might address unintentional firearms injuries in two ways.

First, contemporary firearms can be engineered to reduce injury risk to children. As an example, smart technology that allows only authorized users to pull the trigger of a firearm might prevent unauthorized children or adolescents from shooting unintentionally (14, 15). Sometimes called “personalized guns,” these firearms operate either through wearable technology (e.g., RFID bracelet allows the firearm to be shot) or biometric analysis (e.g., fingerprint recognition).

Second, innovative gun storage options are needed. Some gun owners keep firearms unlocked and loaded so they can use them quickly in case of emergency, such as an intruder in the home. Can we engineer extremely rapid-release safety mechanisms that are low-cost, easy to use, and comfortable for gun owners? Quick-access products currently on the market rely on users to enter codes or biometrics (fingerprints), but most still require 1–2 s to open. Anecdotal evidence suggests many firearm owners find this delay unacceptably long to respond to threats such as a home intruder. With faster openings and modest costs, new firearms storage innovations that appeal to consumers and also improve safety could change the landscape of firearms safety. Recent United States patent filings document early innovations along these lines (16, 17).

## Conclusion

With proper safety measures in place, firearms offer a healthy recreational activity for many people in the United States. It is unrealistic to remove firearms from American homes, but we can take action to reduce unintentional firearms injuries. To do so, we need prioritized, culturally-sensitive and scientifically-motivated public health theory, research, practice, and action.

## Author contributions

DS conceived the idea, prepared and revised the manuscript, and finalized the manuscript.
